# Cost-effectiveness of a short-course antibiotic treatment strategy for the treatment of ventilator-associated pneumonia: an economic analysis of the REGARD-VAP trial

**DOI:** 10.1016/S2214-109X(24)00327-9

**Published:** 2024-11-04

**Authors:** Yiying Cai, Suchart Booraphun, Andrew Yunkai Li, Gyan Kayastha, Paul Anantharajah Tambyah, Ben S Cooper, Nicholas Graves, Yin Mo

**Affiliations:** a Programme in Health Services and Systems Research, Duke-NUS Medical School, National University of Singapore, Singapore; b Sunpasitthiprasong Hospital, Ubon Ratchathani, Thailand; c National University Hospital, Singapore; d Infectious Diseases Translational Research Program, National University of Singapore, Singapore; e Patan Hospital, Patan Academy of Health Sciences, Lalitpur, Nepal; f Centre for Tropical Medicine, Nuffield Department of Medicine, University of Oxford, Oxford, UK; g Mahidol-Oxford Tropical Medicine Research Unit, Faculty of Tropical Medicine, Mahidol University, Bangkok, Thailand

## Abstract

**Background:**

The REGARD-VAP trial showed that individualised shortened antibiotic therapy was non-inferior to usual care for mortality and pneumonia recurrence in patients with ventilator-associated pneumonia (VAP). We aimed to assess the cost-effectiveness of an individualised shortened antibiotic therapy approach in this planned economic analysis.

**Methods:**

REGARD-VAP was a phase 4, multicentre, open-label, randomised trial to assess a short-course antibiotic treatment strategy for treatment of VAP. In this planned economic analysis, we fitted a decision tree with data from the REGARD-VAP trial to estimate the cost-effectiveness of individualised short-course therapy for VAP, compared to usual care from the health system perspective, in Nepal, Singapore, and Thailand. Incremental cost-effectiveness ratios (ICERs) and incremental net monetary benefits with 95% uncertainty intervals (UIs) were reported against relevant willingness-to-pay thresholds. Parameter uncertainties were evaluated using scenario analyses. A value of information analysis was conducted.

**Findings:**

Adopting individualised short-course therapy was cost-effective for Nepal (ICER=US$1086; percentage cost-effectiveness=50·3%), Singapore (ICER=–$6069; percentage cost-effectiveness=55·2%), and Thailand (ICER=$263; percentage cost-effectiveness=60·5%). The associated incremental net monetary benefits were $41 (95% UI –2308 to 2390) in Nepal, $5156 (–45 805 to 56 117) in Singapore, and $804 (–6245 to 7852) in Thailand. Value of information analysis showed that reducing uncertainties for mortality probabilities, bed-day costs, and variable costs were valuable for decision making.

**Interpretation:**

We found that an individualised short-course antibiotics strategy in patients with VAP is likely to be cost-effective in high-income, middle-income, and low-income settings, although with evident uncertainty. Considered alongside the positive externalities of reduced antimicrobial use, our findings foster confidence in policy makers contemplating adoption of short-course antibiotics.

**Funding:**

UK Medical Research Council, Singapore National Medical Research Council, and Wellcome Trust.

## Introduction

Ventilator-associated pneumonia (VAP) is a nosocomial infection associated with poor outcomes. The mainstay management of VAP is antibiotic therapy, but its optimal duration is not known.^1^ Current guidelines recommend 7–8 days according to practices from three randomised trials.^1^, ^2^, ^3^, ^4^ Although the guidelines also suggest shortened duration based on patients' clinical improvement, the approach for identifying patients for shortened duration is not clearly specified.^1^ Given that VAP is a key reason for broad-spectrum-antibiotic prescription in intensive care units (ICUs), and prolonged antibiotic use drives resistance, there is incentive to develop a pragmatic and replicable approach to safely reduce antibiotic duration.

The Reducing Antibiotic Treatment Duration for Ventilator-Associated Pneumonia (REGARD-VAP) trial is a multicentre randomised trial conducted in Nepal, Singapore, and Thailand and is the subject of this analysis.^5^ The authors reported outcomes of an individualised short-course treatment strategy using a set of reproducible clinical criteria where participants were evaluated for fever resolution and haemodynamic stability to individualise antibiotic duration, versus usual practice, in adult patients with VAP.^5^ The trial showed individualised short-course treatment to be non-inferior for the primary outcome of 60-day composite endpoint of death or pneumonia recurrence.^6^ An individualised short-course strategy also reduced overall antibiotic treatment days and antibiotic side-effects, but there were no major differences in length of hospital and ICU stays.^6^

When updating decisions on adoption of a new health intervention, systematic assessment of trade-offs between benefits, harms, and costs is important to maximise population-level health outcomes. Presently, there is no economic evidence on whether short-course antibiotic treatment of VAP is cost-effective when compared to usual care. Shortening antibiotic therapy can reduce antibiotic costs and risk of antibiotic-related adverse events, but any increased incidence in treatment failure or infection recurrence can compromise patient outcomes or increase hospital costs, or both. Even in trials that show small, non-significant differences in adverse events or treatment failures, paradoxical conclusions regarding cost-effectiveness can occasionally be observed if disproportionally high costs were incurred during the episode of treatment failure or adverse event.

**Research in context**

**Evidence before this study**

We searched Embase, MEDLINE (OVID), Web of Science, Cochrane Central Register of Controlled Trials, and ClinicalTrials.gov for economic analyses of randomised controlled trials (RCTs) that assessed antibiotic treatment duration for ventilator-associated pneumonia (VAP), published in English, from database inception to Dec 1, 2023. We used the following search terms: (randomized OR randomised) AND trial AND ventilator AND pneumonia AND (antibiotic OR treatment). Five RCTs were identified and none of these trials assessed the costs or cost-effectiveness of adopting a short-course antibiotic treatment duration for VAP. Previous economic analyses performed for the antibiotic treatment of VAP were based mostly from trials that compared existing versus new antibiotic options.

**Added value of this study**

Our results showed that individualised short-course antibiotic therapy was likely to be cost-effective from the health system perspective in Thailand and Nepal. This finding meant that the health gains produced by individualised short-course antibiotic therapy were likely to be worth the additional costs incurred by these health systems. We also found that individualised short-course antibiotic therapy was likely to be cost-saving to the health system in Singapore. This is the first economic analysis undertaken alongside a pragmatic RCT to assess the cost-effectiveness of a short antibiotic treatment duration in patients with VAP compared to usual care.

**Implications of all the available evidence**

Our results show that adoption of individualised short-course antibiotic treatment duration for VAP is likely to be cost-effective compared with non-adoption in high-income, middle-income, and low-income settings, albeit with notable uncertainty. When considered alongside the potential positive externalities of lowered antimicrobial resistance due to reduced antibiotic use, our results provide some confidence to decision makers that the adoption of an individualised short-course antibiotic strategy to guide antibiotic prescribing in patients with VAP across the various resource settings would be a rational decision from a health system perspective.

In this study, we did a planned economic evaluation of an individualised short-course treatment strategy versus usual care from the perspective of the Nepal, Singapore, and Thailand health-care systems, using findings from the REGARD-VAP trial.

## Methods

### Study design

The REGARD-VAP trial was a multicentre, randomised, open-label trial conducted in 39 ICUs in six hospitals in Nepal, Singapore, and Thailand from May 25**,** 2018, to Dec 16, 2022.^6^ The full protocol is described elsewhere.^5^ Briefly, adult patients with VAP, with a Sepsis-related Organ Failure Assessment score of 10 points or less, not immunocompromised, who had no concurrent infections requiring antibiotic treatment for more than 7 days were enrolled and reviewed daily for fitness criteria to stop antibiotics (ie, defervescence for 48 h and stable blood pressure without inotropic support). In patients randomly assigned to the individualised short-course group, antibiotics administered via all routes (ie, intravenous, oral, and nebulised) were stopped as early as day 3 to day 5 from culture-directed antibiotic initiation (up to 7 days). The exact antibiotic duration was individualised for each patient, depending on when the fitness criteria were met and considering the absence or presence of respiratory cultures.^5^ In the usual care group, all participants received antibiotics for at least 8 days, with the exact duration beyond 8 days decided by treating physicians. The primary outcome was a composite endpoint of death or pneumonia recurrence within 60 days of enrolment. Secondary outcomes were duration of mechanical ventilation, duration of ICU stay and hospitalisation, antibiotic use duration during hospitalisation, readmission to an acute-care hospital, bloodstream infections after being randomly assigned, and acquisition of multidrug resistant infection or colonisation during hospitalisation.

In this economic analysis of the REGARD-VAP trial, we followed the Cost-Effectiveness Analysis alongside Clinical Trials II guidelines.^7^ We developed a decision tree to estimate the cost-effectiveness of an individualised short-course strategy versus usual care strategy from the health-system perspective, using hypothetical cohorts for each of the three participating countries (appendix 1 p 6). Such cohort-based modelling assumes the hypothetical cohort is composed of representative or average individuals in each country, based on inclusion criteria from the REGARD-VAP trial.^6^ The tree showed the unidirectional flow of events from time of VAP diagnosis until death or discharge. There were two strategies: individualised short-course antibiotics and usual care. In patients who were initiated on the individualised short-course strategy, a chance node separated patients into those whose entire antibiotic treatment regimen aligned to the individualised short-course antibiotic strategy as intended, and those that did not. For patients whose antibiotic treatment regimen did not align to the individualised short-course antibiotic strategy, disease progression was assumed to follow that of usual care strategy. In both strategies, chance nodes separated patients into those who were infected with carbapenem-resistant Gram-negative bacilli or not, then patients with and without pneumonia recurrence. There were two possible terminal outcomes: death or discharged alive.

The REGARD-VAP trial protocol, which included this economic evaluation, was approved by the Oxford Tropical Research Ethics Committee and by each countries' relevant ethics committees and regulatory agencies.^5^ All patients provided written informed consent before participation in the trial.

### Model inputs

Model inputs were derived from the REGARD-VAP trial dataset (appendix 1 p 2). Country-specific estimates were derived for probability of mortality, and length of hospital stay from time of VAP diagnosis until death or discharge, based on the per-protocol population of the trial.^6^ The probability of patients who did not receive the complete short-course strategy was estimated using the difference in patient numbers in the intention-to-treat and per-protocol analysis. The number of life-years gained from death averted were estimated by calculating the age-adjusted life expectancy of the modelled cohort using the following formula: life-years gained in a death averted equals the life expectancy of an average person in the country minus the age of the patient at admission.^8^, ^9^, ^10^ This formula assumed that the long-term survival is equivalent to the age-adjusted life expectancy of the country's general population, which is reasonable because it has been shown that development of VAP was not associated with increased mortality risk 1-year post discharge.^11^ We used the life-years gained as the outcome in our base analysis instead of quality-adjusted life-years (QALYs) because the appropriateness of QALYs in evaluation of acute infections such as respiratory tract infections has been disputed. Notably, the ill-health state in acute infections is usually considered transient, which limits the validity of trade-off exercises typically used for utility valuation.^12^, ^13^ Life-years were discounted at 3% per year.^14^

During the primary trial, the following cost categories were collected from time of VAP diagnosis to death or discharge for each patient (or up to 60 days if the patient was not discharged by day 60): bed stay, pharmaceutical products, diagnostic procedures, and consumables for interventional and other procedures (appendix 1 p 2). In Singapore, all costs were provided by hospitals' finance departments. In Thailand and Nepal, bed-stay costs were provided by each hospital's finance department; for all other resource use costs, local researchers estimated the costs using a micro-costing approach, which involves collecting detailed data on resources utilised and their unit costs.^15^ Consistent with a health system perspective, only costs of care incurred by the hospital in delivering care services to the patients were collected. Because we only collected total costs per patient (and not daily costs) for each category, we estimated mean daily cost per category per patient by dividing the total costs from their length of hospital stay after the diagnosis of VAP (or by 60 days, for patients not discharged by day 60). All costs were adjusted to 2022 US$ (1 US$=133 Nepalese rupee, 1 US$=1·3 Singaporean dollar, and 1 US$=35 Thai baht), using the latest gross domestic product (GDP) price deflator data available from the World Bank.^16^ As the time horizon for costs was only for the duration of hospital admission, discounting for costs was not applicable.^14^

### Data analysis

Missing cost data were imputed by use of multiple imputation with chained equations in R and the mice package (version 3.16.0), which assumed that observations were missing at random and could be explained by observed variables.^17^, ^18^ The predictor variables used for imputation were selected based on clinical plausibility, following discussion with the REGARD-VAP trial team (appendix 1 p 4). Predictive mean modelling was used to account for skewed distribution of costs.^17^ Because approximately 10% of cost data were missing, we produced a total of ten imputed datasets and pooled the results using Rubin's rule to obtain a final cost-effectiveness estimate.^19^

Country-specific estimates were derived for probability of mortality and length of stay by use of regression analyses. We used logistic regression to adjust for country-specific probabilities, for the binary outcomes of pneumonia recurrence and death. Tobit regression was used to derive country-specific estimates for mean time to death or discharge. Unlike ordinary least squares regression, which assumes that the data has no ceiling effect, Tobit regression addresses censoring typically found in time-to data and accounted for data censoring beyond 60 days from patient recruitment in the REGARD-VAP trial.^20^ All regression analyses were performed in R, using packages glmnet (version 4.1) and AER (version 1.2).^18^

Economic analysis was done with Microsoft Excel. Incremental cost-effectiveness ratios (ICERs) were calculated as the difference in mean costs between the two strategies, divided by the difference in mean life-years gained.^21^ These differences were compared to a priori defined willingness-to-pay thresholds. If the ICER was less than the willingness-to-pay thresholds, the intervention was likely to be cost-effective; if the ICER was less than zero, then the intervention was likely to be cost-saving. Incremental net monetary benefits of short-course antibiotic strategy were calculated by use of willingness-to-pay thresholds. The willingness-to-pay threshold for Singapore was US$34 615 (45 000 Singaporean dollar) per life-year, based on the government threshold for subsidy of medical technologies.^22^ The willingness-to-pay threshold set by Thailand's Government was US$4751 (160 000 Thai baht) per life-year.^23^ Because there is no established willingness-to-pay threshold for Nepal, we used a willingness-to-pay of 1 × GDP per capita, equivalent to US$1339 (178 000 Nepalese rupee) per life-year.^24^ Probabilistic sensitivity analysis with 1000 iterations was performed using Monte Carlo simulation to generate probability of cost-effectiveness and 95% uncertainty intervals for incremental net monetary benefits. Sampling uncertainties were presented by plotting the distribution of incremental net monetary benefits estimates across the Monte Carlo iterations. Cost-effectiveness acceptability curves were used to show the probability of short-course antibiotic strategy being cost-effective in each country, at plausible willingness-to-pay thresholds between US$0 and US$50 000.

Scenario analyses were undertaken to investigate parameter and imputation uncertainties.^7^ Scenario analyses were done with one-way changes in the following parameters: (1) all patients in the individualised short-course group adhered to the short-course antibiotic regimen, instead of the baseline scenario of where 9% of patients in the individualised short-course group did not adhere to the short-course regimen, (2) not using country-adjusted outcomes, (3) excluding cost of bed stay and treating bed-stay cost as a non-recoverable fixed cost, (4) using QALYs instead of life-years gained, based on health utility weights reported in a previous VAP pharmacoeconomic model,^25^ (5) reduced life expectancy compared to the general population based on a study on patients with sepsis, by Rahmel and colleagues,^26^ (6) using the random forest for multiple imputation of costs, (7) using simple imputation for missing cost data, (8) dropping observations with missing cost data, and (9) using a discount rate of 5% for life-years.

We performed a value of information analysis to estimate the expected value of perfect information (EVPI) and expected value of partial perfect information (EVPPI) for each country.^27^ The EVPI quantified the expected monetary gains of eliminating all uncertainty in all data parameters and represented the upper bound value per patient that can gained from conducting further research to improve estimates in all parameters. The EVPPI represented the upper bound value per patient that can gained from conducting further research to improve the precision of a specific parameter or group of parameters. We estimated EVPPI for the following parameter or group of parameters: probability of adherence to short-course treatment, probability of pneumonia recurrence, probability of infection with carbapenem-resistant Gram-negative bacilli, probability of mortality, length of hospital stays, costs of bed days, variable costs comprising costs of pharmaceutical products, diagnostic procedures, and consumables for interventional and other procedures, and age at admission.

### Role of the funding source

The funders of the study had no role in study design, data collection, data analysis, data interpretation, or writing of the report.

## Results

The results of the REGARD-VAP trial have been published.^6^ Briefly, 460 patients were randomly assigned to the individualised short-course strategy (n=231) or usual care (n=229). 215 (47%) of the 460 patients received combination antibiotics. The median time to culture-directed antibiotic receipt for both the short-course and usual care groups was 0 days (IQR 0 to 2). Within the per-protocol population (n=435), 60-day mortality was observed in 76 (36%) of 211 patients in the short-course strategy group and 87 (39%) of 224 patients in the usual care group (appendix 1 p 5). Pneumonia recurred in 29 (14%) of 211 patients in the short-course strategy group and 30 (13%) of 224 patients in the usual care group. There was no significant difference in the primary composite endpoint of 60-day death or pneumonia recurrence in the intention-to-treat (absolute risk difference –3% [one-sided 95% CI –∞ to 5]) and per-protocol population (absolute risk difference –3% [one-sided 95% CI –∞ to 5]) between the groups. Short-course strategy reduced overall mean antibiotic treatment days during hospitalisation (mean difference 5**·**2 days [95% CI –7·5 to –2·8]), but the lengths of hospital (mean difference –0·45 days [–4·5 to 3·6]) and ICU (mean difference –1·8 days [–6·0 to 2·4]) stays were similar between the groups.

**Figure 1:**
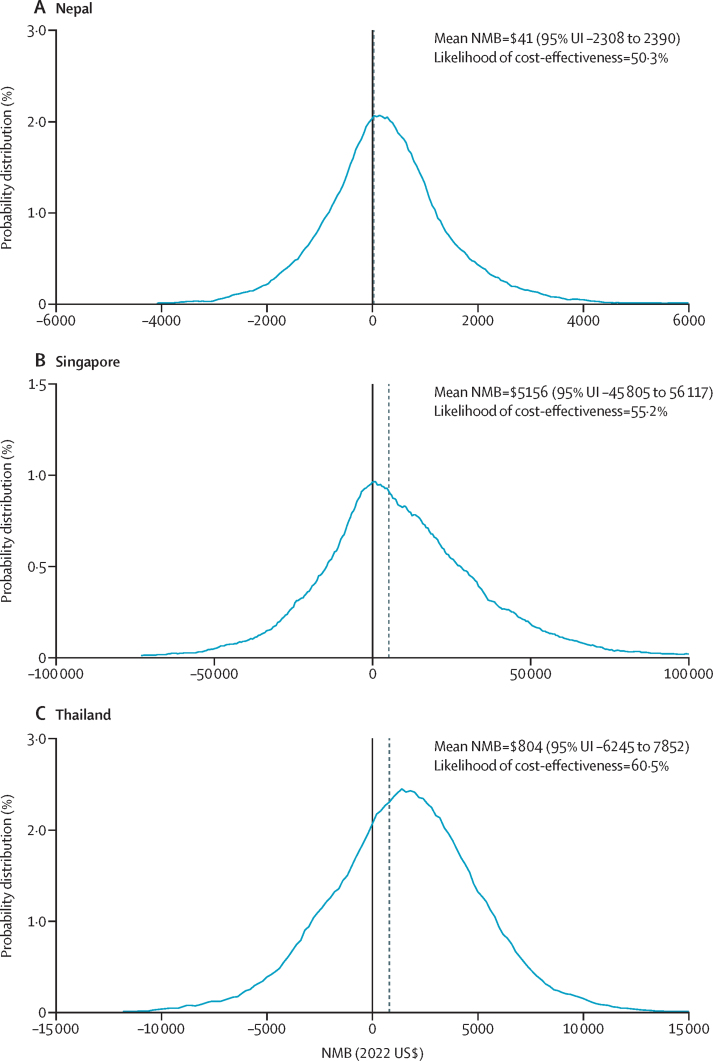
Distribution of NMB from results of the probabilistic sensitivity analysis, based on a priori defined willingness-to-pay thresholds The dashed line represents mean NMB of adopting short-course antibiotics in Nepal (A), Singapore (B), and Thailand (C). NMB=incremental net monetary benefit. UI=uncertainty interval.

**Figure 2:**
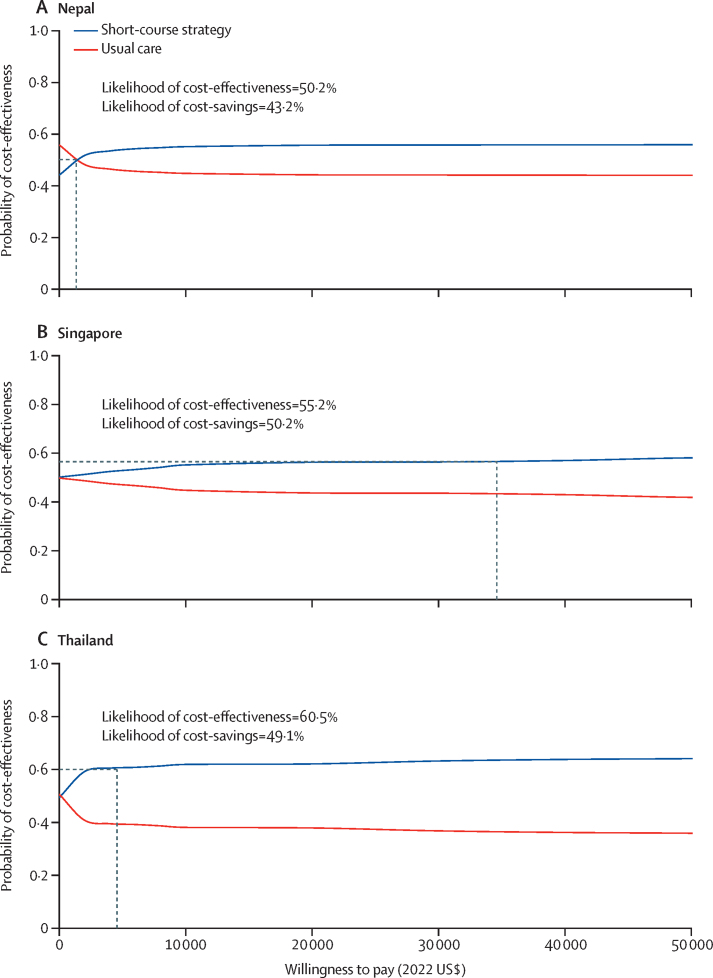
Cost-effectiveness acceptability curves The dashed lines represent the likelihood of cost-effectiveness at each country's willingness to-pay-threshold, in Nepal (A), Singapore (B), and Thailand (C).

The results of the base-case cost-effectiveness analyses are shown in figure 1. In the base-case analyses, incremental net monetary benefits were positive at the predefined willingness-to-pay thresholds for Nepal, Singapore, and Thailand, which meant that short-course strategy was cost-effective compared with usual care from the health system perspective in all three countries (Nepal ICER=$1086, Singapore ICER=–$6069, and Thailand ICER=$263). Mean health-care costs were higher in the short-course strategy compared with usual care for Nepal and Thailand; for Singapore, the mean health-care costs were lower in the short-course strategy than usual care (table 1). Life-years saved were higher in the short-course strategy than in usual care in all three countries (table 1). The cost-effectiveness acceptability curves showed that at the predefined willingness-to-pay thresholds, the likelihood that the short-course strategy was cost-effective in patients with VAP in Nepal was 50·3%, in Singapore was 55·2%, and in Thailand was 60·5% (figure 2). The likelihood that the short-course strategy is cost-saving in Nepal was 43·2%, in Singapore was 50·2%, and in Thailand was 49·1%.

**Table 1:** Base-case analysis of costs, life-years, and ICER per patient

	**Mean costs in 2022 US$ (95% UI)**	**Mean life-years (95% UI)**	ICER*in 2022 US$
**Nepal**			
Short course	1588 (462 to 2713)	4·14 (0·00 to 10·77)	1086
Usual care	1436 (607 to 2264)	4·00 (0·00 to 10·43)	..
Incremental	152 (−1197 to 1501)	0·14 (−1·31 to 1·59)	..
**Singapore**			
Short course	15 575 (655 to 30 496)	9·78 (0·09 to 19·46)	−6069†
Usual care	16 364 (−10 665 to 43 383)	9·65 (0·08 to 19·22)	..
Incremental	−789 (−29 387 to 27 809)	0·13 (−1·09 to 1·34)	..
**Thailand**			
Short course	4586 (230 to 8942)	6·10 (0·00 to 13·52)	263
Usual care	4536 (506 to 8567)	5·92 (0·00 to 13·13)	..
Incremental	50 (−5642 to 5741)	0·19 (−0·71 to 1·09)	..

ICER=incremental cost-effectiveness ratio. UI=uncertainty interval.

* Calculated in accordance with the method by Stinnett and Paltiel.^21^

† Dominant.

In the scenario analyses, one-way parameter change in all modelled scenarios did not increase the ICER beyond the predefined willingness-to-pay thresholds for all countries, and short-course strategy remained likely to be cost-effective in all modelled scenarios (table 2). For Singapore, short-course strategy remained likely to be cost-saving for all modelled scenarios.

**Table 2:** Summary of scenario analyses

	**Mean incremental costs in 2022 US$ (95% UI)**	**Mean incremental life-years (95% UI)**	**Mean incremental net monetary benefit in 2022 US$ (95% UI)**	ICER*in 2022 US$	**Percentage cost-effectiveness at willingness-to-pay threshold**
**Nepal**					
All patients in the individualised short-course group adhered to the short-course antibiotic regimen	156 (−1337 to 1648)	0·16 (−1·41 to 1·74)	64 (−2459 to 2587)	975	51%
Using pooled outcomes for all countries	154 (−1663 to 1971)	0·15 (−0·73 to 1·03)	42 (−2172 to 2256)	1027	53%
Excluding cost of bed stay	35 (−1782 to 1851)	0·19 (−0·69 to 1·06)	213 (−2001 to 2427)	184	56%
Using QALYs gained as outcomes instead of life-years gained	155 (−1200 to 1511)	0·17 (−1·60 to 1·95)	77 (−2633 to 2787)	912	51%
Reduced long-term life expectancy in VAP survivors	145 (−1186 to 1476)	0·13 (−1·09 to 1·35)	33 (−2046 to 2112)	1115	51%
Using random forest for multiple imputation for missing data	158 (−1209 to 1525)	0·16 (−1·29 to 1·60)	53 (−2301 to 2408)	988	52%
Using simple imputation for missing data	166 (−1179 to 1510)	0·21 (−1·58 to 1·99)	111 (−2612 to 2834)	790	52%
Dropping observations with missing data	182 (−1198 to 1561)	0·17 (−1·61 to 1·94)	40 (−2709 to 2790)	1071	50%
Discount rate of 5%	143 (−1200 to 1486)	0·17 (−1·56 to 1·90)	89 (−2555 to 2733)	841	60%
**Singapore**					
All patients in the individualised short-course group adhered to the short-course antibiotic regimen	−956 (−33 235 to 31 326)	0·14 (−1·19 to 1·46)	5687 (−49 931 to 61 303)	−6829†	56%
Using pooled outcomes for all countries	−572 (−21 137 to 19 992)	0·22 (−0·71 to 1·15)	8232 (−30 205 to 46 670)	−2600†	65%
Excluding cost of bed stay	−760 (−26 984 to 25 465)	0·17 (−1·36 to 1·69)	6535 (−52 251 to 65 320)	−4471†	57%
Using QALYs gained as outcomes instead of life-years gained	−833 (−29 798 to 28 133)	0·15 (−1·41 to 1·70)	5992 (−54 678 to 66 662)	−5553†	56%
Reduced long-term life expectancy in VAP survivors	−981 (−30 608 to 28 646)	0·10 (−0·99 to 1·19)	4524 (−43 146 to 52 195)	−9810†	56%
Using random forest for multiple imputation for missing data	−947 (−30 421 to 28 526)	0·13 (−1·09 to 1·35)	5342 (−45 804 to 56 847)	−7285†	57%
Using simple imputation for missing data	−287 (−26 340 to 25 675)	0·11 (−1·48 to 1·70)	3976 (−56 790 to 64 742)	−2609†	54%
Dropping observations with missing data	−1871 (−34 574 to 30 832)	0·13 (−1·43 to 1·69)	6475 (−55 781 to 68 730)	−14 392†	58%
Discount rate of 5%	−1039 (−30 724 to 28 646)	0·17 (−1·38 to 1·72)	6998 (−54 806 to 68 802)	−6112†	57%
**Thailand**					
All patients in the individualised short-course group adhered to the short-course antibiotic regimen	64 (−6086 to 6213)	0·20 (−0·75 to 1·16)	866 (−6729 to 8462)	320	60%
Using pooled outcomes for all countries	44 (−6066 to 6093)	0·20 (−0·66 to 1·05)	854 (−6372 to 8080)	220	60%
Excluding cost of bed stay	54 (−5463 to 5572)	0·25 (−0·91 to 1·40)	1082 (−6523 to 8688)	216	61%
Using QALYs gained as outcomes instead of life-years gained	87 (−5679 to 5853)	0·20 (−0·78 to 1·18)	816 (−6580 to 8212)	435	59%
Reduced long-term life expectancy in VAP survivors	9 (−5749 to 5767)	0·16 (−0·63 to 0·95)	732 (−6122 to 7587)	56	59%
Using random forest for multiple imputation for missing data	73 (−5593 to 5739)	0·20 (−0·71 to 1·10)	832 (−6167 to 7830)	365	59%
Using simple imputation for missing data	93 (−5390 to 5576)	0·24 (−0·93 to 1·41)	1001 (−6699 to 8701)	388	61%
Dropping observations with missing data	145 (−5248 to 5538)	0·25 (−0·94 to 1·44)	998 (−6610 to 8606)	580	58%
Discount rate of 5%	70 (−5644 to 5784)	0·25 (−0·90 to 1·39)	1057 (−6570 to 8864)	280	61%

ICER=incremental cost-effectiveness ratio. QALYs=quality-adjusted life-years. UI=uncertainty interval. VAP=ventilator-associated pneumonia.

* Calculated in accordance with the method by Stinnett and Paltiel.^21^

† Dominant.

**Figure 3:**
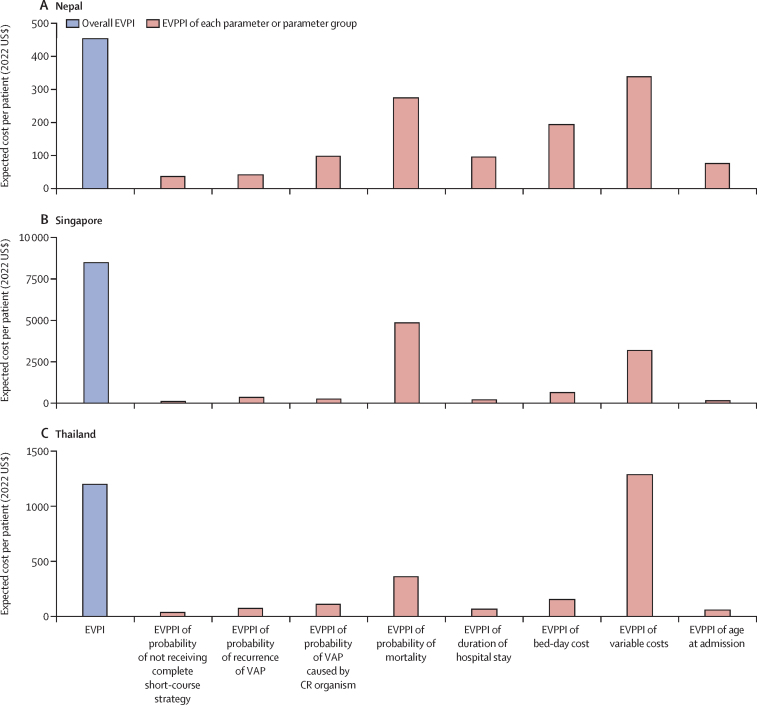
Value of information analysis CR=carbapenem-resistant. EVPI=expected value of perfect information. EVPPI=expected value of partial perfect information. VAP=ventilator-associated pneumonia.

In our value of information analysis, the EVPI per patient in Nepal was $454, in Singapore was $8455, and in Thailand was $1196. Although all parameters or group of parameters contributed to model uncertainties, the parameters or group of parameters that contributed most to parameter uncertainties in all three countries were the probabilities of mortality, costs of bed days, and variable costs (comprised of costs of pharmaceutical products, diagnostic procedures, and consumables for interventional and other procedures; figure 3).

## Discussion

Compared with usual care, the decision to adopt an individualised short-course antibiotic strategy using a set of reproducible clinical criteria in patients with VAP is likely to be cost-effective from the health system perspective in Nepal, Singapore, and Thailand, albeit with substantial uncertainty. In a high-income setting such as Singapore, adoption of short-course strategy is also likely to be cost-saving to the health system. Our findings are robust to the scenario analyses. The value of information analysis revealed the greatest value for decision making would arise from reducing uncertainties for mortality risk, costs of bed days, and variable costs.

From a population-level health policy standpoint, results from clinical studies are usually insufficient to guide the adoption of a new health intervention, because multiple factors beyond efficacy go into such decisions. Our cost-effectiveness analysis combined relevant short-term and long-term aspects of patients' costs and outcomes to strengthen the evidence for rational decision making on short-course individualised therapy in VAP. Because the composite endpoint used in the REGARD-VAP trial was unsuitable for economic analyses, we used a decision tree to present endpoints of pneumonia recurrence and patient mortality in a disaggregated form to compare costs and outcomes across the strategies.^7^ Our estimates are conservative because our model did not consider the potential spill-over benefits of reduced antibiotic use for reducing resistance carriage and onward transmission of multidrug resistant organisms within the hospital, which would probably result in additional gains in health outcomes and monetary savings. In a previous meta-analysis, Mo and colleagues^28^ found that 1 day of additional antibiotic treatment was associated with a 7% absolute increase in risk of resistance carriage, when antibiotics administered were not effective against the resistance phenotype in the colonising bacteria carriage. Additionally, we adopted a lower bound of the 1–3 times GDP per capita suggested by WHO for use in low-income and middle-income countries for Nepal because a threshold has not yet been formally established.^29^

Our study has other strengths. First, previous cost-effectiveness analyses in VAP were derived from trials that were mostly undertaken in conjunction with new product launches and were restricted to high-income settings.^30^ Such trials usually have limited external validity due to restrictive inclusion and exclusion criteria, artificially enhanced compliance, and use comparators that do not reflect usual clinical practice. In comparison, our cost-effectiveness analysis was based on the REGARD-VAP trial, which evaluated a set of simple and reproducible clinical criteria to guide antibiotic duration against real-world practice conditions. Such a pragmatic trial design is considered optimal for cost-effectiveness studies because it confers high external validity to the findings.^7^ Second, we derived cost-effectiveness estimates for each country using country-specific costs for resource use and adjusting for country effects for outcomes.^5^ This approach was used because the health systems design, cost structure, and resource utilisation are likely to substantially differ between the countries, and a trial-wide cost-effectiveness estimate based on overall trial costs and outcomes would be unlikely to be useful to any of the countries that participated in the REGARD-VAP trial.

As expected, we observed substantial disparity in costs and outcomes between Nepal, Singapore, and Thailand, probably due to differences in clinical practice patterns and health-care costs. In Thailand, the local practice of discharging patients with poor prognoses to their homes probably contributed to the shorter hospital stay compared with Nepal and Singapore, which in turn impacted cost estimates of overall bed stay. Similarly, higher costs of pharmaceutical products in Singapore meant that any shortening of antibiotic duration probably resulted in substantial savings to offset costs due to any increased incidence of treatment failure or pneumonia recurrence, which can explain why short-course individualised antibiotics are more likely to be cost-saving in Singapore, but not in Thailand or Nepal.

Our study has limitations. First, we estimated mean daily costs in each patient by dividing the total costs by their length of stay after VAP. Given that resource use often peaks in the earlier days of a hospital stay and tapers off in the later days, use of mean costs could have resulted in overestimation of actual savings from any reduced hospital length of stay. Additionally, the complex clinical manifestations of VAP meant that we could only tabulate all resource-use costs instead of VAP-specific costs. Although we do not expect non-VAP resource use costs to differ substantially between the groups due to randomisation, the limitations in our cost estimation methods probably contributed to the high variability in our costs estimates, which might explain the findings in our value of information analysis. Second, although costs associated with the increased adverse effects noted in the usual care group in the primary trial (eg, testing or treatment for *Clostridioides difficile* diarrhoea) would have been accounted for because we tabulated all resource-use costs after VAP onset, we did not separately quantify these costs in our model. Hence, information regarding potential cost savings from reduced adverse effects in the short-course antibiotic strategy could not be separately discerned in our analysis. Third, all bed-day costs were based on costs provided by each hospital's accounting department. Because accounting costs are designed for expenditure recovery, they would inevitably include overhead expenses and might not truly represent the forgone cost of the next best alternative of the bed day (ie, opportunity cost). Lastly, in keeping with the original trial, our model only applied to the subset of patients with VAP with clinical stability, no concurrent infections, and who were not immunocompromised. While these clinical criteria probably represent a large proportion of patients with VAP, our findings cannot be applied to immunocompromised patients or those with concurrent infections.

Prudent antibiotic use is needed to preserve our current antibiotic armamentarium. We found that an individualised short-course antibiotic strategy in patients with VAP is likely to be cost-effective across low-income, middle-income, and high-income settings from the health system perspective, compared with usual practice. The data from our analysis, alongside findings from the REGARD-VAP trial, strengthen the evidence for decision makers looking to incorporate the individualised short-course antibiotic strategy in suitable patients when developing national or institutional antibiotic prescribing policies in patients with VAP.

### Contributors

### Equitable partnership declaration

### Data sharing

Data collected for the study will be made available to others upon request to the corresponding author, following the Mahidol-Oxford Research Unit's data sharing policy.

## Declaration of interests

We declare no competing interests.

## Supplementary Materials

Supplementary appendix 1

Equitable Partnership Declaration

## References

[1] Kalil AC, Metersky ML, Klompas M (2016). Management of adults with hospital-acquired and ventilator-associated pneumonia: 2016 clinical practice guidelines by the Infectious Diseases Society of America and the American Thoracic Society. Clin Infect Dis.

[2] Kollef MH, Chastre J, Clavel M (2012). A randomized trial of 7-day doripenem versus 10-day imipenem-cilastatin for ventilator-associated pneumonia. Crit Care.

[3] Chastre J, Wolff M, Fagon JY (2003). Comparison of 8 vs 15 days of antibiotic therapy for ventilator-associated pneumonia in adults: a randomized trial. JAMA.

[4] Capellier G, Mockly H, Charpentier C (2012). Early-onset ventilator-associated pneumonia in adults randomized clinical trial: comparison of 8 versus 15 days of antibiotic treatment. PLoS One.

[5] Mo Y, West TE, MacLaren G (2021). Reducing antibiotic treatment duration for ventilator-associated pneumonia (REGARD-VAP): a trial protocol for a randomised clinical trial. BMJ Open.

[6] Mo Y, Booraphun S, Li AY (2024). Individualised, short-course antibiotic treatment versus usual long-course treatment for ventilator-associated pneumonia (REGARD-VAP): a multicentre, individually randomised, open-label, non-inferiority trial. Lancet Respir Med.

[7] Ramsey SD, Willke RJ, Glick H (2015). Cost-effectiveness analysis alongside clinical trials II—an ISPOR Good Research Practices Task Force report. Value Health.

[8] World Bank Group (2022). Life expectancy at birth, total (years) - Nepal. https://data.worldbank.org/indicator/SP.DYN.LE00.IN?locations=NP.

[9] Department of Statistics Singapore (2023). Death and life expectancy. https://www.singstat.gov.sg/find-data/search-by-theme/population/death-and-life-expectancy/latest-data.

[10] World Bank Group (2022). Life expectancy at birth, total (years) - Thailand. https://data.worldbank.org/indicator/SP.DYN.LE00.IN?locations=TH.

[11] Mergulhão P, Pereira JG, Fernandes AV (2024). Epidemiology and burden of ventilator-associated pneumonia among adult intensive care unit patients: a Portuguese, multicenter, retrospective study (eVAP-PT Study). Antibiotics (Basel).

[12] Holmes EAF, Hughes DA (2019). Challenges for economic evaluation of health care strategies to contain antimicrobial resistance. Antibiotics (Basel).

[13] Bala MV, Zarkin GA (2000). Are QALYs an appropriate measure for valuing morbidity in acute diseases?. Health Econ.

[14] Attema AE, Brouwer WBF, Claxton K (2018). Discounting in economic evaluations. PharmacoEconomics.

[15] Hendriks ME, Kundu P, Boers AC (2014). Step-by-step guideline for disease-specific costing studies in low- and middle-income countries: a mixed methodology. Glob Health Action.

[16] World Bank Group (2023). Inflation, GDP deflator (annual %). https://data.worldbank.org/indicator/NY.GDP.DEFL.KD.ZG.

[17] Van Buuren S, Oudshoorn CG (2000).

[18] R Core Team (2022). R: a language and environment for statistical computing. https://www.R-project.org/.

[19] Rubin DB (1987).

[20] Foster G, Kalenkoski CM (2013). Tobit or OLS? An empirical evaluation under different diary window lengths. Appl Econ.

[21] Stinnett AA, Paltiel AD (1997). Estimating CE ratios under second-order uncertainty: the mean ratio versus the ratio of means. Med Decis Making.

[22] Ministry of Health Singapore (2022). Medical technologies evaluation methods and process guide. https://www.ace-hta.gov.sg/docs/default-source/process-methods/ace-methods-and-process-guide-for-drug-evaluation-(20-dec-2019).pdf.

[23] Nimdet K, Ngorsuraches S (2015). Willingness to pay per quality-adjusted life year for life-saving treatments in Thailand. BMJ Open.

[24] World Bank Group (2022). GDP per capita (current US$) - Nepal. https://data.worldbank.org/indicator/NY.GDP.PCAP.CD?locations=NP.

[25] Grau S, Alvarez-Lerma F, del Castillo A, Neipp R, Rubio-Terrés C (2005). Cost-effectiveness analysis of the treatment of ventilator-associated pneumonia with linezolid or vancomycin in Spain. J Chemother.

[26] Rahmel T, Schmitz S, Nowak H (2020). Long-term mortality and outcome in hospital survivors of septic shock, sepsis, and severe infections: the importance of aftercare. PLoS One.

[27] Oostenbrink JB, Al MJ, Oppe M, Rutten-van Mölken MP (2008). Expected value of perfect information: an empirical example of reducing decision uncertainty by conducting additional research. Value Health.

[28] Mo Y, Oonsivilai M, Lim C, Niehus R, Cooper BS (2023). Implications of reducing antibiotic treatment duration for antimicrobial resistance in hospital settings: a modelling study and meta-analysis. PLoS Med.

[29] Woods B, Revill P, Sculpher M, Claxton K (2016). Country-level cost-effectiveness thresholds: initial estimates and the need for further research. Value Health.

[30] Wagner AP, Enne VI, Livermore DM, Craig JV, Turner DA (2020). Review of health economic models exploring and evaluating treatment and management of hospital-acquired pneumonia and ventilator-associated pneumonia. J Hosp Infect.

