# Supplementary material for: Cost-effectiveness of a short-course antibiotic treatment strategy for the treatment of ventilator-associated pneumonia: an economic analysis of the REGARD-VAP trial
Source: Lancet Glob Health. 2024 Nov 4;12(12):e2059–67. doi: 10.1016/S2214-109X(24)00327-9 (PMC11579304; doi:10.1016/S2214-109X(24)00327-9)
Supplement: Supplementary appendix 1 [file mmc1.pdf]

THE LANCET
Global Health

Supplementary appendix 1

This appendix formed part of the original submission and has been peer reviewed. We post it as supplied by the authors.

Supplement to: Cai Y, Booraphun S, Li AY, et al. Cost-effectiveness of a short-course antibiotic treatment strategy for the treatment of ventilator-associated pneumonia: an economic analysis of the REGARD-VAP trial. *Lancet Glob Health* 2024; published online Nov 4. https://doi.org/10.1016/S2214-109X(24)00327-9.

SUPPLEMENTARY APPENDIX

Table of contents

Supplementary Table 1. Input parameters for decision tree 2

Supplementary Table 2. Predictor variables for multiple imputation of missing cost data....... 4

Supplementary Table 3. Summary of baseline characteristics and outcomes of the per-protocol population from the REGARD-VAP trial 5

Supplementary Figure 1. Structure of the decision tree depicting the two comparator groups 6

Supplementary Figure 2. Example of data distributions used in the economic model 7

References 8

Supplementary Table 1. Input parameters for decision tree

Parameters[a]	Based on REGARD-VAP trial results	Adjusted for each country			Distribution	Reference
		Nepal	Singapore	Thailand		
**Probabilities – short-course strategy**						
Non-adherence to short-course strategy	0·09 (0·02)	0·09 (0·02)	0·09 (0·02)	0·09 (0·02)	Beta	
VAP due to CR GNB	0·34 (0·03)	0·34 (0·03)	0·34 (0·03)	0·34 (0·03)	Beta	Derived from
Pneumonia recurrence in patients with CR GNB	0·29 (0·05)	0·29 (0·05)	0·29 (0·05)	0·29 (0·05)	Beta	the
Pneumonia recurrence in patients with non-CR GNB	0·04 (0·03)	0·04 (0·03)	0·04 (0·03)	0·04 (0·03)	Beta	REGARD-
Mortality						VAP dataset[1]
CR GNB with pneumonia recurrence	0·63 (0·07)	0·70 (0·09)	0·33 (0·12)	0·64 (0·07)	Beta	
CR GNB with no pneumonia recurrence	0·43 (0·05)	0·51 (0·09)	0·18 (0·07)	0·44 (0·05)	Beta	
Non-CR GNB with pneumonia recurrence	0·45 (0·08)	0·56 (0·12)	0·21 (0·09)	0·49 (0·09)	Beta	
Non-CR GNB with no pneumonia recurrence	0·27 (0·03)	0·36 (0·08)	0·11 (0·04)	0·30 (0·04)	Beta	
**Probabilities – standard of care strategy**						
VAP due to CR GNB	0·34 (0·03)	0·34 (0·03)	0·34 (0·03)	0·34 (0·03)	Beta	Derived from
Pneumonia recurrence in patients with CR GNB	0·29 (0·05)	0·29 (0·05)	0·29 (0·05)	0·29 (0·05)	Beta	the
Pneumonia recurrence in patients with non-CR GNB	0·04 (0·03)	0·04 (0·03)	0·04 (0·03)	0·04 (0·03)	Beta	REGARD-
Mortality						VAP dataset[1]
CR GNB, pneumonia recurrence	0·65 (0·07)	0·72 (0·09)	0·35 (0·12)	0·66 (0·07)	Beta	
CR GNB, no pneumonia recurrence	0·46 (0·05)	0·53 (0·09)	0·19 (0·08)	0·46 (0·05)	Beta	
Non-CR GNB, pneumonia recurrence	0·48 (0·09)	0·59 (0·12)	0·23 (0·10)	0·52 (0·09)	Beta	
Non-CR GNB, no pneumonia recurrence	0·29 (0·04)	0·38 (0·09)	0·11 (0·05)	0·32 (0·04)	Beta	
**Hospital length of stay in days after VAP – short course strategy**						
CR GNB, pneumonia recurrence, death	36 (3)	40 (4)	46 (4)	35 (3)	Gamma	Derived from
CR GNB, pneumonia recurrence, no death	41 (3)	45 (4)	50 (4)	40 (3)	Gamma	the
CR GNB, no pneumonia recurrence, death	29 (2)	33 (3)	39 (4)	28 (2)	Gamma	REGARD-
CR GNB, no pneumonia recurrence, no death	35 (2)	38 (3)	43 (3)	33 (2)	Gamma	VAP dataset[1]
Non-CR GNB, pneumonia recurrence, death	18 (3)	22 (4)	28 (4)	17 (3)	Gamma	
Non-CR GNB, pneumonia recurrence, no death	24 (3)	27 (4)	32 (4)	22 (3)	Gamma	
Non-CR GNB, no pneumonia recurrence, death	12 (2)	16 (3)	21 (3)	10 (2)	Gamma	
Non-CR GNB, no pneumonia recurrence, no death	17 (2)	20 (3)	26 (3)	15 (2)	Gamma	
**Hospital length of stay in days after VAP – standard of care strategy**						
CR GNB, pneumonia recurrence, death	35 (3)	40 (4)	45 (4)	34 (3)	Gamma	Derived from
CR GNB, pneumonia recurrence, no death	40 (3)	44 (4)	50 (4)	39 (3)	Gamma	the
CR GNB, no pneumonia recurrence, death	28 (2)	33 (3)	38 (4)	28 (2)	Gamma	REGARD-
CR GNB, no pneumonia recurrence, no death	34 (2)	37 (3)	43 (3)	32 (2)	Gamma	VAP dataset[1]
Non-CR GNB, pneumonia recurrence, death	17 (3)	22 (4)	27 (4)	17 (3)	Gamma	
Non-CR GNB, pneumonia recurrence, no death	23 (3)	26 (5)	32 (4)	21 (3)	Gamma	
Non-CR GNB, no pneumonia recurrence, death	11 (2)	15 (2)	20 (3)	10 (2)	Gamma	
Non-CR GNB, no pneumonia recurrence, no death	17 (2)	20 (3)	25 (3)	14 (2)	Gamma	
**Costs per day – Short course strategy[b]**						
Bed-stay	-	17 (14)	112 (106)	23 (10)	Gamma	Derived from
Pharmaceutical products	-	10 (6)	67 (84)	11 (10)	Gamma	the
Diagnostic procedures	-	19 (16)	91 (99)	54 (50)	Gamma	

Interventional procedures	-	16 (8)	207 (183)	128 (99)	Gamma	REGARD-VAP dataset[1]
**Costs per day – Standard course strategy**[b]						
Bed-stay	-	12 (5)	123 (155)	27 (32)	Gamma	Derived from
Pharmaceutical products	-	9 (8)	97 (189)	12 (9)	Gamma	the
Diagnostic procedures	-	18 (12)	54 (50)	61 (50)	Gamma	REGARD-
Interventional procedures	-	18 (8)	230 (354)	122 (78)	Gamma	VAP dataset[1]
**Other parameters**						
Utility weight of patients discharged alive post-VAP[c]	-	0.63 – 0.92	0.63 – 0.92	0.63 – 0.92	Uniform	[2]
Mean age at admission	61 (17)	61 (17)	61 (17)	61 (17)	Gamma	[1]
Life expectancy of general population	-	71	83	78	Fixed	[3-5]
Hazard of death in sepsis patients[d]	-	1.71 (0.01)	1.71 (0.01)	1.71 (0.01)	Log-normal	[6]
Willingness-to-pay	-	1,339	34,615	4,571	Fixed	[7-9]

[a] Values are presented as mean and standard error
[b] Based on multiple imputation using predictive mean matching.
[c] Not applied in base-analysis. Utility weight was only applied in Scenario IV of the scenario analysis.
[d] Not applied in base-analysis. Hazard of death in sepsis patients was only applied in Scenario V of the scenario analysis.

Abbreviations used in Supplementary Table 1. CR, carbapenem-resistant; GNB, Gram-negative bacteria; SD, standard deviation; VAP, ventilator-associated pneumonia

Supplementary Table 2. Predictor variables for multiple imputation of missing cost data

Data group	Predictor variables included for imputing missing cost data
Demographics	Country, age at admission
Clinical characteristics	Intervention group, presence of carbapenem-resistant infection,
Outcomes	Length of hospital stay, mortality, presence of pneumonia recurrence

Supplementary Table 3. Summary of baseline characteristics and outcomes of the per-protocol population from the REGARD-VAP trial

Characteristics [1]	Individualised short-course (n = 211)	Usual care (n = 224)
Baseline characteristics		
Median age in years (IQR)	63 (50 – 73)	66 (51 – 74)
Gender		
Female	85 (40%)	82 (37%)
Male	126 (60%)	142 (63%)
Country of enrolment		
Nepal	19 (9%)	19 (8%)
Singapore	23 (11%)	24 (10%)
Thailand	169 (80%)	181(81%)
Type of ICU		
Medical	65 (31%)	72 (32%)
Surgical	146 (69%)	152 (68%)
Infection and treatment characteristics		
Median SOFA score (IQR)	6 (4 – 8)	6 (4 – 7)
CR bacteria grown in respiratory culture during index VAP episode	64 (30%)	65 (29%)
Median time in days between VAP symptoms onset to culture-directed antibiotics (IQR)	0 (0 – 2)	0 (0 – 2)
Proportion of patients who received culture-directed antibiotics from symptom onset	115 (55%)	119 (53%)
Outcomes		
Composite outcome of mortality and pneumonia recurrence	87 (41%)	99 (44%)
Mortality	76 (36%)	87 (39%)
Pneumonia recurrence	29 (14%)	30 (13%)
Median antibiotic duration for index VAP episode in days (IQR)	6 (5 – 7)	14 (10 – 21)
Mean (SD) duration of ICU admission in days	27 (24)	29 (24)
Any antibiotic side effects	17 (8%)	86 (38%)

Abbreviations used in Supplementary Table 3. CR, carbapenem-resistant; ICU, intensive care unit; IQR, interquartile range; SD, standard deviation; VAP, ventilator-associated pneumonia

Supplementary Figure 1. Structure of the decision tree depicting the two comparator groups

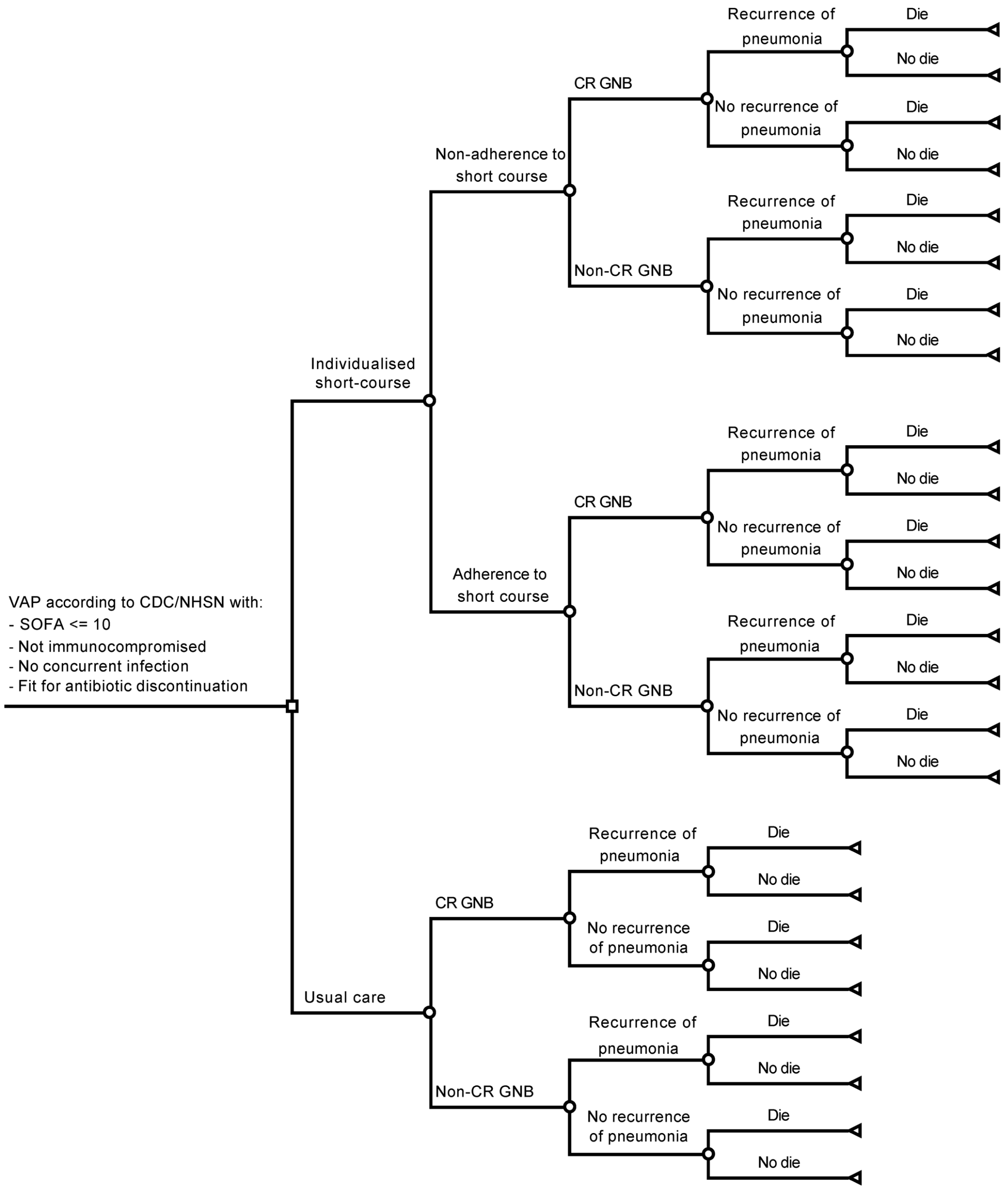

Supplementary Figure 2. Example of data distributions used in the economic model

(a) Beta distribution for probabilities with mean probability = 0.63 and standard deviation = 0.07

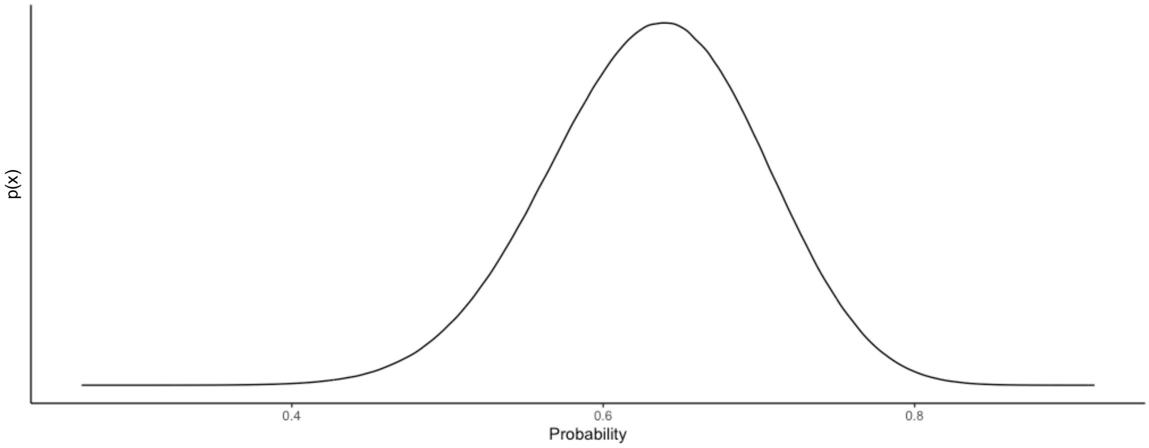

(b) Gamma distribution for costs with mean cost = US$17 and standard deviation = US$14

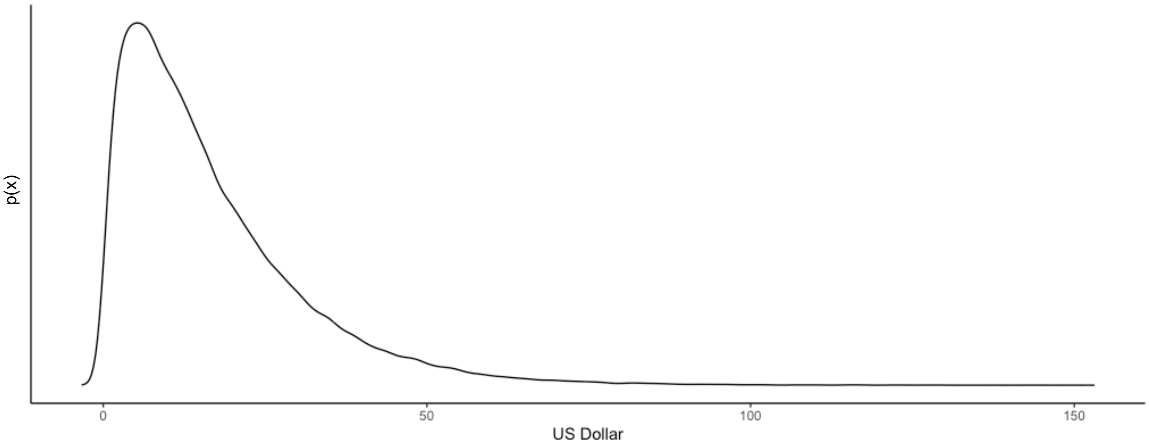

(c) Uniform distribution for QALY ranging from minimum = 0.63 and maximum = 0.92

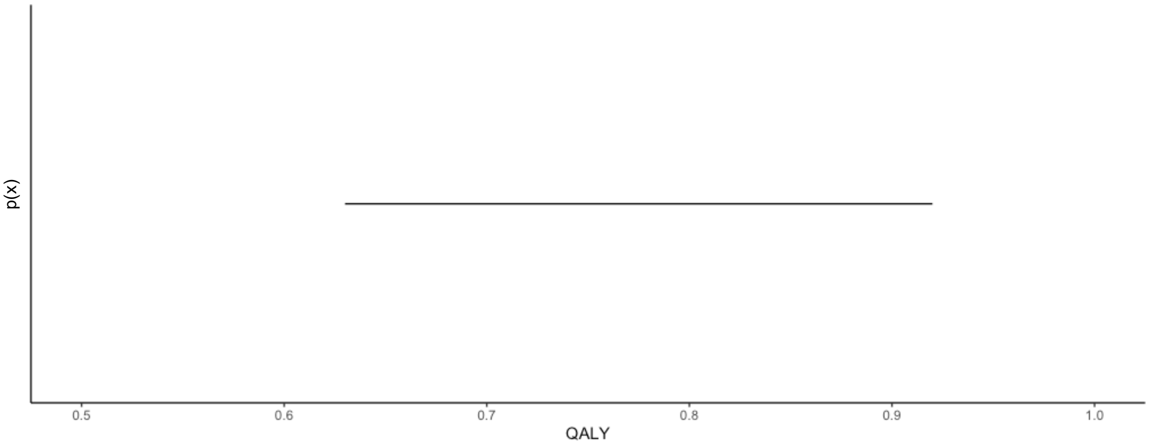

References

1. Mo Y, Booraphun S, Li AY, et al. Individualised, short-course antibiotic treatment versus usual long-course treatment for ventilator-associated pneumonia (REGARD-VAP): a multicentre, individually randomised, open-label, non-inferiority trial. *Lancet Respir Med* 2024.

2. Grau S, Alvarez-Lerma F, del Castillo A, Neipp R, Rubio-Terrés C. Cost-effectiveness analysis of the treatment of ventilator-associated pneumonia with linezolid or vancomycin in Spain. *J Chemother* 2005; **17**(2): 203-11.

3. World Bank Group. Life expectancy at birth, total (years) - Nepal. 2022. https://data.worldbank.org/indicator/SP.DYN.LE00.IN?locations=NP (accessed 10/11/2023.

4. Department of Statistics SIngapore. Death and Life Expectancy. 2023. https://www.singstat.gov.sg/find-data/search-by-theme/population/death-and-life-expectancy/latest-data (accessed 8th March 2023.

5. World Bank Group. Life expectancy at birth, total (years) - Thailand. 2022. https://data.worldbank.org/indicator/SP.DYN.LE00.IN?locations=TH (accessed 10/11/2023.

6. Rahmel T, Schmitz S, Nowak H, et al. Long-term mortality and outcome in hospital survivors of septic shock, sepsis, and severe infections: The importance of aftercare. *PLoS One* 2020; **15**(2): e0228952.

7. Ministry of Health Singapore. MEDICAL TECHNOLOGIES EVALUATION METHODS AND PROCESS GUIDE. 2022.

8. Nimdet K, Ngorsuraches S. Willingness to pay per quality-adjusted life year for life-saving treatments in Thailand. *BMJ Open* 2015; **5**(10): e008123.

9. World Bank Group. GDP per capita (current US$) - Nepal. 2022. https://data.worldbank.org/indicator/NY.GDP.PCAP.CD?locations=NP (accessed 10/11/2023.
