# Supplementary material for: Cost-effectiveness of a short-course antibiotic treatment strategy for the treatment of ventilator-associated pneumonia: an economic analysis of the REGARD-VAP trial
Source: Lancet Glob Health. 2024 Nov 4;12(12):e2059–67. doi: 10.1016/S2214-109X(24)00327-9 (PMC11579304; doi:10.1016/S2214-109X(24)00327-9)
Supplement: Equitable Partnership Declaration [file mmc2.pdf]

THE LANCET
Global Health

Supplementary appendix 2

This Equitable Partnership Declaration (EPD) was submitted by the authors, and we reproduce it as supplied. It has not been peer reviewed. *The Lancet*'s editorial processes have not been applied to the EPD.

Supplement to: Cai Y, Booraphun S, Li AY, et al. Cost-effectiveness of a short-course antibiotic treatment strategy for the treatment of ventilator-associated pneumonia: an economic analysis of the REGARD-VAP trial. *Lancet Glob Health* 2024; published online Nov 4. https://doi.org/10.1016/S2214-109X(24)00327-9.

Equitable Partnership Declaration questions

Researcher considerations

1. Please detail the involvement that researchers who are based in the region(s) of study had during a) study design; b) clinical study processes, such as processing blood samples, prescribing medication, or patient recruitment; c) data interpretation; and d) manuscript preparation, commenting on all aspects. If they were not involved in any of these aspects, please explain why.

   This question is intended for international partnerships; if all your authors are based in the area of study, this question is not applicable.

   This should include a thorough description of their leadership role(s) in the study. Are local researchers named in the author list or the acknowledgements, or are they not mentioned at all (and, if not, why)? Please also describe the involvement of early career researchers based in the location of the study. Some of this information might be repeated from the Contributors section in the manuscript. Note: we adhere to [ICMJE authorship criteria] when deciding who should be named on a paper.

**a) Study design:**<br>BS Cooper, PA Tambyah, N Graves, and Y Mo conceptualised the study. Y Cai, N Graves, BS Cooper, and Y Mo designed the health economics study.
**b) Clinical study processes:**<br>Patient recruitment and data collection were performed by S Booraphun, AY Li, and G Kayastha.
**c) Data interpretation:**<br>Data analysis and interpretation was performed by Y Cai, N Graves and Y Mo, who directly accessed and verified underlying data reported in the manuscript
**d) Manuscript preparation:**<br>Y Cai prepared the first manuscript draft. All authors reviewed the manuscript for important intellectual content, and approved the submission of the current version.

2. Were the data used in your study collected by authors named on the paper, or have they been extracted from a source such as a national survey? ie, is this a secondary analysis of data that were not collected by the authors of this paper. If the authors of this paper were not involved in data collection, how were data interpreted with sufficient contextual knowledge?

   The Lancet Global Health *believe contextual understanding is crucial for informed data analysis and interpretation.*

The data used in the study were collected by the authors named on the paper.

3. How was funding used to remunerate and enhance the skills of researchers and institutions based in the area(s) of study? And how was funding used to improve research infrastructure in the area of study?

   Potentially effective investments into long-term skills and opportunities within institutions could include training or mentorship in analytical techniques and manuscript writing, opportunities to lead all or specific aspects of the study, financial remuneration rather than requiring volunteers, and other professional development and educational opportunities.

   Improvements to research infrastructure could be funding of extended trial designs (such as platform trials) and use of master protocols to enable these designs, establishment of long-term contracts for research staff, building research facilities, and local control of funding allocation.

**Skills:**<br>Over 200 personals were involved in the REGARD-VAP study including the health economic component. These included clinicians, research coordinators and laboratory technicians. They received Good Clinical Practice training, basic research methodology training and were involved in regular audits with immediate feedback on data quality. Selected members also travelled to Singapore, Vietnam and Thailand for courses and hospital visits.
**Research infrastructure:**<br>REGARD-VAP trial was a major trial undertaken by the various research offices and units in the respective study sites. The study offered funding for equipment e.g. freezers, DNA extraction kits etc, and helped to build local research teams.

4. How did you safeguard the researchers who implemented the study?

   Please describe how you guaranteed safe working conditions for study staff, including provision of appropriate personal protective equipment, protection from violence, and prevention of overworking.

The project manager met with the local site teams on a weekly basis to ensure safety of the teams, especially during the COVID-19 pandemic. The core team provided personal protective equipment and infection prevention and control guidance. The respective research teams were given opportunities at least at an annual basis to provide feedback on the core managing team.

<u>Benefits to the communities and regions of study</u>

5. How does the study address the research and policy priorities of its location?

   How were the local priorities determined and then used to inform the research question? Who decided which priorities to take forward? Which elements of the study address those priorities?

Ventilator associated pneumonia and extended antibiotic use are highly prevalent in countries where the study was conducted. The local investigators were heavily involved in the protocol development process. We also performed a qualitative study concurrently with this trial, which

found that engagement of the policy makers is essential at ensuring the trial findings are implemented widely. This was an additional motivation for our health economic evaluation.

6. How will research products be shared in the community of study?

For instance, will you be providing written or oral layperson summaries for non-academic information sharing? Will study data be made available to institutions in the region(s) of study? The Lancet Global Health *encourages authors to translate the summary (abstract) into relevant languages after paper editing; do you intend to translate your summary?*

We will share the manuscript (including abstract in native languages) with the local sites, ethics boards and healthcare authorities. We will also share the key findings through our clinical trial network newsletters and social media.

7. How were individuals, communities, and environments protected from harm?

a) How did you ensure that sensitive patient data was handled safely and respectfully? Was there any potential for stigma or discrimination against participants arising from any of the procedures or outcomes of the study?

All data were anonymised.

b) Might any of the tests be experienced as invasive or culturally insensitive?

NA

c) How did you determine that work was sensitive to traditions, restrictions, and considerations of all cultural and religious groups in the study population?

NA

d) Were biowaste and radioactive waste disposed of in accordance with local laws?

NA

e) Were any structures built that would have impacted members of the community or the environment (such as handwashing facilities in a public space)? If so, how did you ensure that you had appropriate community buy-in?

NA

f) How might the study have impacted existing health-care resources (such as staff workloads, use of equipment that is typically employed elsewhere, or reallocation of public funds)?

The study provided adequate funding to local teams to ensure manageable workload, and also paid for equipment which were lacking.

8. Finally, please provide the title (eg, Dr/Prof, Mr/Mrs/Ms/Mx), name, and email address of an author who can be contacted about this statement. This can be the corresponding author.

**Name:** Mo Yin<br>**Email: mdcmy@nus.edu.sg**
